# Barriers and facilitators to nationwide implementation of the malaria vaccine in Ghana

**DOI:** 10.1093/heapol/czac077

**Published:** 2022-09-09

**Authors:** Omolola Oyinkan Adeshina, Solomon Nyame, James Milner, Ai Milojevic, Kwaku Poku Asante

**Affiliations:** Department of Public Health, Environments and Society, London School of Hygiene & Tropical Medicine, 15-17 Tavistock Place, London WC1H 9SH, UK; Kintampo Health Research Centre, Ghana Health Service, P.O. Box 200, Kintampo North Municipality, Ghana; Department of Public Health, Environments and Society, London School of Hygiene & Tropical Medicine, 15-17 Tavistock Place, London WC1H 9SH, UK; Centre on Climate Change and Planetary Health, London School of Hygiene & Tropical Medicine, Keppel Street, London WC1E 7HT, UK; Department of Public Health, Environments and Society, London School of Hygiene & Tropical Medicine, 15-17 Tavistock Place, London WC1H 9SH, UK; Centre on Climate Change and Planetary Health, London School of Hygiene & Tropical Medicine, Keppel Street, London WC1E 7HT, UK; Kintampo Health Research Centre, Ghana Health Service, P.O. Box 200, Kintampo North Municipality, Ghana; Department of Disease Control, London School of Hygiene & Tropical Medicine, Keppel Street, London WC1E 7HT, UK

**Keywords:** Malaria, RTS, S/AS01E malaria vaccine, children under 5 years, force field analysis, pilot, implementation programme, Ghana, qualitative research

## Abstract

Interventions such as antimalarial drugs, bed nets and insecticides have helped curb the burden of malaria in the past decade, yet malaria remains a leading cause of morbidity and mortality in children below the age of 5 years. In 2019, Ghana, Malawi and Kenya in sub-Saharan Africa (countries with moderate to high transmission areas of malaria and deaths) started piloting the RTS,S/AS01E malaria vaccine in selected regions. Using qualitative methods, this study examined the main factors (forces) that will influence or hinder the nationwide implementation of the malaria vaccine, if approved, in Ghana. We conducted in-depth interviews with 12 key individuals (national, research/academia and programme implementing partners) in the public health sector in Ghana from October 2018 to February 2019. Results were analysed using Kurt Lewin’s force field analysis to understand how organizations interact with their external environment in the delivery of health policies such as the implementation of the malaria vaccine. We found that the disease burden of malaria deaths in Ghana, the efficacy of the vaccine, stakeholder involvement and evidence for the feasibility of vaccine delivery generated by the consortium of researchers (body of researchers) that can track the implementation were the driving forces to scale up the vaccine into a routine health system. On the other hand, the needed logistics, funding, administration of the four-dose vaccine and follow-up were identified as potential barriers. The most influential force collectively highlighted by the respondents was the disease burden, and the most influential barrier was the logistics of delivering the vaccine. Our findings provide decision makers with key barriers and facilitators to guide policy and decision-making for malaria control in Ghana and other similar settings in low- and middle-income countries.

Key messages

- After Malaria Vaccine Pilot Implementation Project, the most influential driving forces of Ghana’s decision for nationwide scale-up of the RTS,S/AS01E malaria vaccine are the malaria disease burden and the efficacy/effectiveness of the vaccine.
- The main potential barriers to the scale-up of the RTS,S/AS01E malaria vaccine are the logistics for the nationwide delivery of the vaccine and funding.
- The evidence-based data currently being generated and gathered during the malaria vaccine project can be used for decision-making to scale up the nationwide implementation of the vaccine.

## Introduction

Malaria is a burden of disease for many countries, especially in sub-Saharan Africa (SSA) (van den Berg *et al.*, 2019). As of 2018, malaria has led to an estimated 219 million cases in over 80 countries (Choi *et al.*, 2019). In Ghana, *Plasmodium falciparum* is the primary malaria parasite causing nearly 2000 deaths annually and the malaria parasite affecting 48% of children under the age of 5 years (Asante *et al.*, 2016). These cases in children led to an estimated 30% of hospital admissions are due to malaria (Aregawi *et al.*, 2017), and ∼20–30% of malaria cases are severe, predominantly in children under the age of 5 years and pregnant women (Yankson *et al.*, 2019).

In 2004, Ghana adopted the use of artesunate–amodiaquine for the treatment of uncomplicated malaria; however, in 2014, artemether–lumefantrine and dihydroartemisinin-piperaquine were included for individuals unable to tolerate artesunate–amodiaquine (GSS, 2020; MOH, 2020). These three first-line drugs, a type of artemisinin-based combination therapy (ACT), were selected based on evidence-based data in regard to efficacy and safety in Africa (Owusu-Agyei *et al.*, 2008). The Ghana Malaria Indicator Survey (GMIS) reports that 48% of children under the age of 5 years with recent fever received an ACT in 2008 and this percentage increased to 85% in 2019. This survey also reports that 67% of households have access to insecticide-treated nets and that 43% of children under 5 years of age use insecticide-treated nets (GSS, 2020). Although Ghana has made progress in malaria prevention and control since the 1990s, malaria still remains a public health burden that requires additional measures to combat it (van den Berg *et al.*, 2019).

In 2016, WHO issued a position paper for interested countries in the Malaria Vaccine Pilot Implementation Project (MVIP) of the RTS,S/AS01E malaria vaccine, of which Ghana showed interest (MOH, 2016). The RTS,S/AS01E malaria vaccine is designed to prevent the *P. falciparum species* from infecting red blood cells. The vaccine has been developed over a 30-year process and is the most advanced vaccine candidate that could contribute to malaria control globally, as well as the first human parasite vaccine that has the potential to prevent malaria cases (Agnandji *et al.*, 2015; Morrison, 2015). Before Ghana’s selection to the MVIP in 2017 (Laurens, 2020), Ghana participated in RTS,S/AS01E Phase 3 clinical trials that showed the efficacy and safety of the vaccine. In the Phase 3 clinical trials, children aged 6 weeks to 17 months received four doses of the vaccine at specific time intervals: 0, 1 and 2 months and a booster dose at 20 months (RTS, 2015; Asante *et al.*, 2016). The vaccine was found to reduce the burden of clinical and severe malaria by ∼40% when used together with other malaria preventive measures, such as bed nets and insecticides (Agnandji *et al.*, 2014).

The MVIP in Ghana commenced in May 2019, shortly after the launch of the vaccine in Malawi in April 2019 (Bell *et al.*, 2020; Hogan *et al.*, 2020). Alongside Ghana and Malawi, Kenya is also one of the chosen countries in SSA for the MVIP (Hogan *et al.*, 2020). These three countries were selected out of 10 African countries. The selection was based on experience with clinical trials of the malaria vaccine and the robustness of their in-country immunization programmes (Adepoju, 2019). Nationwide roll-out of the vaccine in Ghana would occur through the Expanded Programme on Immunization (EPI), which is responsible for all vaccine roll-out nationwide (MOH/GHS, 2014). Since the establishment of the National Malaria Control Programme (NMCP) in the late 1950s, Ghana has made significant progress in control interventions for malaria (Awine *et al.*, 2017; GHS, 2015). The 2019 GMIS indicates that 90% of caregivers would be willing to have their children vaccinated with the malaria vaccine (GSS, 2020).

This study explored facilitators and barriers to the roll-out process of the malaria vaccine, rather than behavioural factors among the public. Facilitators and barriers have also been identified in previous vaccines introduced in Ghana. For example, between 2008 and 2014, there were challenges with the administration, follow-up and delivery for the third dose of the pentavalent vaccine that led to a significant decrease in uptake. Similarly, in 2012, a second dose of measles-containing vaccine was also introduced, but coverage has remained low (Yawson *et al.*, 2017). Nevertheless, the rotavirus and pneumococcal vaccines introduced in Ghana were successful owing to factors such as the effectiveness of the vaccine and stakeholder involvement (Donkor *et al.*, 2013; Enweronu-Laryea *et al.*, 2014b). However, the recent pandemic brought about the development of multiple COVID-19 vaccines. Although these vaccines have been effective, it has led to an increase in vaccine hesitancy globally (Tagoe *et al.*, 2021). The introduction of new vaccines comes with its drivers and challenges, and the RTS,S/AS01E malaria vaccine may be no different. To help scale up the malaria vaccine nationwide, there is an urgent need to identify factors that could facilitate or act as barriers to the scale-up. At the conclusion of the ongoing MVIP phase in 2023, the Ghana Health Service (GHS) can either choose to support or not support the nationwide introduction of the RTS,S/AS01E malaria vaccine (IFPMA, 2022).

## Materials and methods

### Force field analysis framework

A force field analysis (FFA) framework, developed by social psychologist Kurt Lewin in the 1940s, can be used to identify factors (forces) that lead to a change that is capable of shaping policy processes (Lewin, 1947). In recent years, the framework has been adapted for management, policy situations and health promotion (MacDuffie and DePoy, 2004; URC, 2008; Capatina *et al.*, 2017). Lewin’s framework looks at forces that facilitate movement towards a goal (driving forces) or that hinder movement towards a goal (restraining forces) (Lewin, 1947; Cathro, 2011). According to Lewin’s framework, before the change, both opposing forces are said to be in equilibrium. For change to happen, driving forces need to be capitalized on before equilibrium can be reached that is favourable to the situation at hand. The FFA framework has been utilized in fields such as medicine, nursing and software development, all of which have focused on the implementation of change (MacDuffie and DePoy, 2004; URC, 2008; Cathro, 2011; Capatina *et al.*, 2017).

The principle of the FFA framework requires the understanding of a process through (1) the identification of driving factors, (2) the identification of opposing factors, (3) the ranking of the importance or impact of the different factors and (4) the development of an action plan for change (Lewin, 1947; Cathro, 2011; Capatina *et al.*, 2017). This study applied the FFA framework to assess driving forces and barriers in relation to the scale-up of the malaria vaccine into the routine health system in Ghana through in-depth interviews (IDIs) using a qualitative method.

### Data collection

Based on stakeholder mapping (BSR, 2011), we identified eight institutions in Ghana and examined how relevant these institutions are to the eventual scale-up of the malaria vaccine. Institutions were selected that were likely to have a high level of interest and influence on the malaria vaccine scale-up and would be able to bring about change to address the burden of malaria in Ghana. Within these institutions, 12 key individuals were selected based on affiliation with the GHS, international nongovernmental organizations (that have made contributions to the previous phases of the development of the malaria vaccine), members of academic and multilateral agencies (responsible for the scale-up) (MOH, 2016). We used a combination of purposive and snowballing sampling techniques considering the unique nature of the respondents. Table 1 highlights the characteristics of respondents.

**Table 1.** Characteristics of respondents

Characteristics of respondents	*n* (%)
Gender	
Male	11 (91.6)
Female	1 (8.3)
Numbers of years at institution	
<15 years	6 (50)
>15 years	6 (50)
Number of years involved in malaria treatment initiatives	
<15 years	3 (25)
>15 years	9 (75)
Number of years involved on malaria vaccine development	
<15 years	3 (25)
>15 years	9 (75)

These respondents were selected to participate in interviews conducted in person or by phone (10 interviews in person and 2 via phone call). Interview guides were developed and pre-tested before IDIs were conducted between October 2018 and February 2019, with interviews lasting from 30 min to 1 h. All interviews were digitally recorded and transcribed verbatim with participants anonymized, and written notes were taken. In total, four GHS employees, five external personnel and three medical research personnel were interviewed. GHS personnel were from the following institutions: NMCP, EPI and the authors’ institute. External personnel were from WHO, Food and Drugs Authority (FDA) and Program for Appropriate Technology in Health (PATH). Medical research personnel were from the Noguchi Memorial Institute for Medical Research and the University of Ghana. Information from the interviews was analysed and extracted in accordance with the FFA framework (Baulcomb, 2003). Information from interview quotes was illustrated thematically.

Based on recommendations from respondents during the interviews, we obtained information on relevant forces for the FFA framework (Baulcomb, 2003). We also identified and assessed relevant documents that respondents referred to during the interviews, including evidence-based reports on the vaccine, immunization reports, policy, strategic framework, vaccine presentation reports, vaccine materials and organizational reports. There were 48 documents in total, which included 3 Ministry of Health reports, 6 GHS reports, 15 WHO reports and 23 publications.

### Data analysis

**Figure 1.**
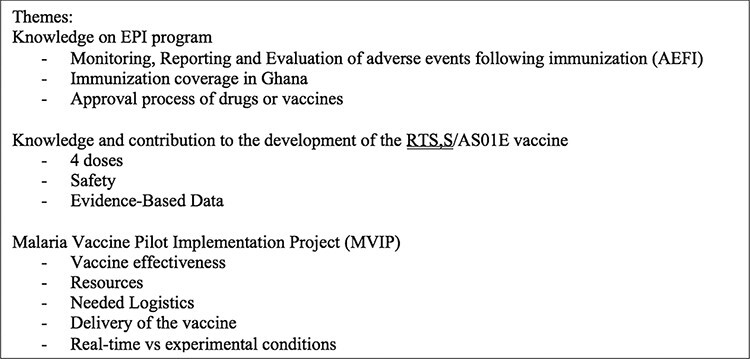
Coding frame for data analysis

**Figure 2.**
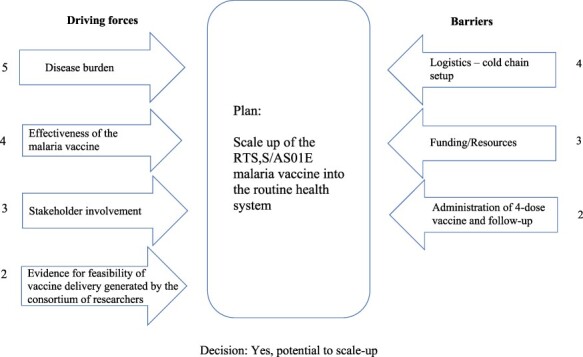
FFA to scale up the vaccine into the routine health system. This figure illustrates the forces in support of the change to scale up the vaccine and forces against the change on a scale of 1–5, i.e., 1 = extremely weak and 5 = extremely strong

Data for transcripts from the IDIs were imported into NVivo 12 QSR International for Mac (QSRInternational, 2020). The data were extracted and analysed thematically by two researchers (O.O.A. and S.N.). Figure 1 highlights the coding frame used for the analysis, which was developed by considering the study objectives, literature on the vaccine and review of transcripts. Three main themes and 11 sub-themes were used. The theme under the MVIP was focused on the enablers and barriers pertaining to vaccine effectiveness, resources, needed logistics, delivery of the vaccine and real-time vs experimental conditions. A topic guide was developed, and coding themes were identified in the following categories: immunization, types of vaccines under EPI, malaria vaccine, implementation and policy. These themes were used to identify patterns of communication in the documents and to synthesize analysed data concerning evidence, stakeholder and other information on the vaccine. Table 2 depicts number of respondents that identified forces to influence or hinder the scale-up of the malaria vaccine in Figure 2.

**Table 2.** Number of respondents that identified forces to influence and hinder the scale-up of the RTS,S/AS01E malaria vaccine

Identified forces to influence and hinder the scale-up of the RTS,S/AS01E malaria vaccine	No. of respondents
Driving forces	
Disease burden	9
Effectiveness of the malaria vaccine	8
Stakeholder involvement	6
Evidence for feasibility of vaccine delivery generated by consortium of researchers	4
Restraining forces	
Logistics—cold-chain set-up	6
Funding/resources	5
Administration of four-dose vaccine and follow-up	4

## Results

Four driving forces were identified: the disease burden; effectiveness of the malaria vaccine; stakeholder involvement and evidence for the feasibility of vaccine delivery generated by the consortium of researchers. Conversely, three barriers were identified equally that might be an obstacle to the scale-up (Figure 2).

### Driving forces that will positively influence the MVIP

#### Disease burden

Based on respondents’ ratings of forces that would influence the scale-up (Table 2), the most pivotal driving force that emerged from data transcription was reducing the burden of malaria deaths on the healthcare system (Figure 2). Respondents highlighted the cost to the system and the need to reduce the disease burden as a driver, because if the malaria vaccine can reduce the disease burden, then it is worth implementing. During the IDI, one interviewee remarked that:

The vaccine is not replacing other malaria interventions but is meant to add on. The research has shown that it is able to reduce this disease burden and deaths due to malaria. Of course, this is within the context of other interventions so it is seen as something the EPI should implement because malaria is the number one killer of children under-fives in the country. There is also the issue of resistance to drugs, we felt if there is an alternative that can help to reduce the burden and numbers of those who get the disease then it is better for us and for our population. (Participant 6)

#### Effectiveness of the malaria vaccine

The effectiveness of the vaccine is of public relevance because a malarial infection can lead to mortality and comorbidities:

For instance, for about 15 years, since 2003, we have not encountered any death from measles in the country and it is all due to the success of vaccinations and the immunization programme. The malaria vaccine is seen as an effective way of controlling the disease, reducing deaths, especially if you are to achieve reduction of under-fives deaths then this is a tool which is very much needed. (Participant 7)

#### Stakeholder involvement

Based on the assessment of recommended documents, all vaccines introduced in Ghana thus far have involved stakeholders at various levels, and most vaccines have been implemented first outside the country before being implemented in Ghana (Figure 2):

The MVIP may not have been necessary considering there was a positive opinion from the European Medicines Agency that reviewed the data that was generated. Other vaccines introduced in Ghana have not gone through this phase, but this is the first malaria vaccine, so lots of precaution is required. (Participant 10)

Several groups and bodies have contributed thus far to the RTS,S/AS01E malaria vaccine development and implementation process, especially the EPI for the scale-up:

It is a multi-partner process so we at EPI deploy the vaccines. This malaria vaccine must be available, safe, and effective. The government and other teams doing their evaluations are also involved which means other parties’ collective agreement is needed for a nationwide implementation. We have adapted training materials and modified our involvement. We have a NITAG established that will review and advise for the reason why we should put the vaccine onto the EPI programme. (Participant 4)

The National Immunization Technical Advisory Group (NITAG) was established in 2018. It is made up of experts, members of the Ministry of Health-GHS, other ministries, departments and agencies. The NITAG provides evidence-informed advice to inform vaccines and immunization processes:

In the *NITAG* we have to come up with a document to show that this is useful for it to go into policy and this is why this group was formed. The minister inaugurated the group based on each individual’s capacity and capability, not on where they are coming from. So I am an epidemiologist; there is a virologist, a pharmacist … It is based on one’s individual knowledge and skills, so we will advise technically. It should be evidence-based. We need to have the evidence to show to the government that yes, based on 1, 2, 3, 4, 5, the government can go ahead and accept it. (Participant 2)

Besides the recently established NITAG, a Technical Working Group (TWG) had already been established in 2009. Both groups are working with the policy, planning, monitoring and evaluation division of the Ministry of Health-GHS and WHO consultants to ensure the adequate information gathering to share with the government:

When discussions of the malaria vaccine were ongoing back in 2009, a TWG was formed. The idea of the TWG was that somewhere along the line we may have to introduce the malaria vaccine. There was a need to start looking at how we will introduce it to the EPI programme, so we had several meetings and thereafter people were assigned to evaluate the programme, the safety and all those things. All this was done and as time went on, it became obvious that the vaccine will have to be introduced. At that time, we also got to know that Glaxo (GSK) had produced a vaccine … We are now using the Mosquirix which will be introduced by Glaxo (GSK). There are others on the market, but they have not been made available or gotten to the stage where people can use. (Participant 4)

Nonetheless, within the EPI, there have been positive experiences of implementing new vaccines in Ghana, especially with involving parents or the caregivers in the process:

To us, our experience with new vaccines has been very good but then it needs the preparatory activities to train the staff, to inform the communities, to educate them, so they know about the vaccine. (Participant 6)

The nationwide implementation process of any vaccine is lengthy and requires sufficient dissemination of information among involved groups and bodies. A current TWG member and a former EPI and WHO representative at the country level remarked:

Recently, the WHO sent a consultant to take us through a document on the stages each vaccine has to go through. We will need to provide this information to the government and not by word of mouth. It is a long process and normally these policies get sent to the policy, planning, monitoring and evaluation division of the ministry for review, before lawyers review too and so on … It takes quite some time. (Participant 8)

#### Evidence for the feasibility of vaccine delivery generated by the consortium of researchers

Analysis of key documents confirmed most vaccines introduced in Ghana have been tested outside the country; in contrast, a consortium of researchers in-country partners in Ghana, Kenya and Malawi to carry out evaluation studies for the MVIP: feasibility group, safety group and impact group. This consortium of researchers made up of expert researchers and advocates on malaria was formed in 2009. The evidence for the feasibility of vaccine delivery generated by the consortium of researchers represents the fourth driving force. Providing policy makers with evidence-based data on safety measures, pharmacovigilance, close monitoring of children receiving the vaccine and assessing feasibility will help with scale-up of the malaria vaccine nationwide. Among this consortium of researchers, the EPI is responsible for vaccine delivery and uptake during the pilot stage and will be involved in the scale-up of the RTS,S/AS01E malaria vaccine if approved.

Interviews with respondents revealed that the feasibility assessment from the consortium of researchers will have an influence on the delivery of the RTS,S/AS01E vaccine. The delivery schedule for the RTS,S/AS01E malaria vaccine is at 6, 7, 9 and 24 months. With the current immunization schedule in Ghana (MOH, 2016), at 6 months of age, children take only vitamin A so the malaria vaccine will be added to this. At 7 months, no vaccines are currently taken so the malaria vaccine will be the only one given. At 9 months, measles–rubella plus the malaria vaccine will be given, and at 24 months, only the malaria vaccine will be given. This is potentially beneficial because the delivery of the vaccine provides an avenue for children and their caregivers to have additional contacts with the healthcare delivery set-up.

### Restraining forces that will negatively influence the MVIP

Three barriers were identified: logistics—cold-chain set-up, funding/resources and administration of four-dose vaccine and follow-up (Figure 2).

#### Logistics—cold-chain set-up

The most pivotal barrier identified in Figure 2 was the logistics, particularly in relation to the cold-chain set-up. For example, the additional storage facilities required to maintain the cold chain until the child receives the RTS,S/AS01E vaccine. An interviewee highlighted that:

Additional storage facilities are required. This means that at the headquarters in Accra [capital city of Ghana] where the vaccines will arrive, there should be enough storage. Before vaccines are moved from the capital of Ghana to the other regions. So, movement from Accra to Ho, the regional capital of Volta region, and movement from Accra to Cape coast in the Central region, to Sunyani in the Brong-Ahafo region are issues that need to be considered. (Participant 2)

Hence, there needs to be a proper and adequate cold-chain set-up for scale-up.

#### Funding/resources

In the past decade, Ghana has been receiving support from the public–private partnerships (WHO, the World Bank, the Bill and Melinda Gates Foundation, GAVI, etc.). Respondents indicated that support from the Global Alliance for Vaccines and Immunization (GAVI) could change in the coming years, leading to the second barrier (funding/resources) (Figure 2). Interviewees expressed concern for further funding resources being needed to complete the MVIP before consideration of a scale-up:

It is also not certain whether there will be adequate funding to complete the pilot programme. The funds … where do we get it if GAVI decides to stop supporting the Ghanaian government. This means that Ghana will have to buy all its vaccines and will not be receiving the support of GAVI if this happens. This can affect the course of the pilot to policy process. (Participant 3)

#### Administration of four-dose vaccine and follow-up

The third barrier identified was administration of the four-dose vaccine and follow-up related to vaccinating in real-life (Figure 2). Interviewees perceived the feasibility of rolling out the RTS,S/AS01E vaccine in real-life will be similar to the pilot because the RTS,S/AS01E vaccine’s four-dose schedule does fall within the standard immunization schedule in Ghana except the last dose (which is given at 24 months). Moreover, the RTS,S/AS01E vaccine is one of the first vaccines on the EPI to be administered out of schedule. Thus, adequate infrastructure will be needed to deliver the four doses and for children to receive each dose with little or no dropout. Children in Ghana are currently taking 13 antigens, not including the malaria vaccine. The addition of the RTS,S/AS01E malaria vaccine will increase this to 14. An interviewee said:

It is an issue because this vaccine will be given as an injection and when you look at the vaccines that the children are already being vaccinated with; it is an issue. You see, when you look at it from that angle, compliance is going to be challenging, the mothers will be complaining, one injection too many. This may not be well received with some caregivers. (Participant 8)

In the Phase 3 trials of the vaccine, the coverage of the four doses was high but may be lower under routine implementation, as remarked by a respondent:

The EPI will be leading in ensuring that the vaccines get to these regions to vaccinate the children who will qualify … If children took the vaccine and did not get a booster or a repeat dose later, then what we noticed was that the protection of the vaccine quickly went down. What this means is that, if they are within the first 5 years, like 2 years, 3 years or 4 years and do not get a repeat of this vaccine then that under aged 5 protection will not be there. But we also noticed that if you took a repeat of the vaccine, then the protection went up again. (Participant 9)

From the interviews, the MVIP is important to know if the four doses can be delivered effectively within Ghana’s routine immunization schedule effectively, including responding to unanswered safety questions or doubts on the vaccine for potential scale-up.

## Discussion

Malaria continues to be one of the leading causes of morbidity and mortality in Ghana, particularly among children under 5 years. Thus, there are efforts to identify and implement effective strategies to curb the burden of malaria in Ghana. These strategies include effort to introduce the malaria vaccine. Evidence shows that the malaria vaccine provides 40% partial protectivity of the malaria vaccine in children, matching results from Phase 2 and 3 trials (RTS, 2014; Geleijnse, 2015; Asante *et al.*, 2016). This study sought to identify the barriers and facilitators for the possible scale-up of the malaria vaccine. The findings of this study highlighted factors that are likely to influence stakeholders of the GHS on the nationwide roll-out of the RTS,S/AS01E malaria vaccine and barriers that may constrain the malaria vaccine policy process. The driving forces identified were the disease burden of malaria, effectiveness of the malaria vaccine, stakeholder involvement and evidence for the feasibility of vaccine delivery generated by the consortium of researchers.

The process of highlighting the main driver and the effectiveness of the malaria vaccine is necessary to scale up. However, there need to be sufficient resources available for the scale-up, clear communication channels, stakeholder involvement and proper cold-chain set-up. Funding from GAVI is dependent on if Ghana moves from an upper- and middle-income country to a lower- and middle-income country (MOH/GHS, 2014). Additionally, the budgetary implications of scaling up the vaccine may likely include the cost of the vaccine and the expansion of the cold-chain set-up. The malaria vaccine will have to be introduced in selected regions in Ghana before scaling up in all 16 regions in the country (GhanaMissionUN, 2021). Moreover, a collaboration of various stakeholders at the international, in-country level of participating countries and a consortium of researchers of the MVIP are needed continuously to facilitate the scale-up of the malaria vaccine. In terms of the administration of the vaccine and follow-up under experimental conditions, health workers were responsible for bringing children with their caregivers to health facilities. However, in the scale-up, the onus will be on caregivers, so measures will need to be put in place to ensure caregivers are able to bring children to health facilities for vaccination. In the vaccine development, Phase 2 and 3 trials of the malaria vaccine were controlled, so outcomes were predicted based on the testing and follow-up of participants. However, the MVIP is being conducted in the context of routine use and so extraneous factors cannot be controlled, such as ensuring children follow-up with health facilities for the fourth dose (Asante *et al.*, 2019).

The large disease burden of malaria as a driving factor for the roll-out of the vaccine is similar to when the GHS introduced the rotavirus vaccine and pneumococcal vaccines simultaneously in 2012 to combat pneumonia and severe infant diarrhoea (Donkor *et al.*, 2013; Enweronu-Laryea *et al.*, 2014a). Similarly, other countries, such as Kenya and South Africa, also introduced the rotavirus vaccine and pneumococcal vaccines based on disease burden and reduced mortality in children aged 5 years and below (Muendo *et al.*, 2018; Kleynhans *et al.*, 2021). However, vaccines like that of polio with a relatively uncommon disease burden have been introduced globally (Cochi and Pallansch, 2021). The malaria vaccine will aid in fighting malaria and is intended to be in addition to, rather than a replacement for, other malaria interventions such as insecticide-treated nets and insecticide sprays (Winskill *et al.*, 2019). Research has shown through modelling data that the vaccine can reduce the disease burden and deaths due to malaria within the context of other interventions (Penny *et al.*, 2015; 2016). It can be argued that it is worth implementing the malaria vaccine if it can reduce the burden of malaria in the country as observed in the Phase 3 trials and modelled data. (RTS, 2014; Penny *et al.*, 2015; 2016).

Although the effectiveness of the malaria vaccine is relatively low at 40% partial protective in children, it has nonetheless been introduced (RTS, 2014; Geleijnse, 2015; Asante *et al.*, 2016). This is comparable to the Bacillus Calmette–Guérin (BCG) vaccine implementation in Ghana and Kenya when the efficacy was 56%. Today, the BCG vaccine is accepted globally (Hogan *et al.*, 2020; Adesanya *et al.*, 2021). For previous vaccines introduced in Ghana, the EPI has strengthened its communication to fit the introduction of each vaccine and has shown that acceptance of a new vaccine is dependent on public education and adequate dissemination of evidence (MOH/GHS, 2014). Similar findings were demonstrated through the introduction of the pneumococcal vaccine in the Gambia (Roca *et al.*, 2011) and polio vaccine in Nigeria (Ozawa and Stack, 2013). GHS stakeholders involved in the MVIP are continuously collaborating within its operating environment with bodies such as the EPI and externally with WHO, PATH, the FDA, academic institutions and international government agencies evaluating medicinal products to facilitate the introduction of the RTS,S/AS01E malaria vaccine. These stakeholders are attempting to increase awareness of evidence-based data for the remaining phases of the MVIP and to identify and resolve concerns from the consortium of researchers (Siegelaub, 2005; Folayan *et al.*, 2019). Consequently, there is ongoing stakeholder involvement to enhance transparency, communication and involvement, and to ensure sociocultural sensitivity of disseminated information between the bodies involved and caregivers of children who will receive the vaccine (Coleman *et al.*, 2019).

The main barriers we identified were the cold-chain set-up, funding and administration of the four-dose vaccine and follow-up. The findings of these three aforementioned barriers for the roll-out of the vaccine are similar to those of the meningococcal serogroup A conjugate vaccine (MACV) introduced in 2017 onto the EPI in Burkina Faso. The MACV introduction in Burkina Faso was faced with poor cold-chain infrastructure, inadequate supply of vaccines from one region to another, the cold-chain logistics training of staff in the maintenance of the vaccine and insufficient funding for carrying out vaccination activities (Nkwenkeu *et al.*, 2020). Improving the cold-chain logistics and acquiring the financial resources were needed for transporting the MACV vaccine at both the district and regional levels and carrying out vaccination activities. The roll-out of the RTS,S/AS01E in Ghana will also require an expanded cold-chain set-up and funding for vaccination activities. In addition, the introduction of the MACV in Burkina Faso experienced administration and follow-up issues because the opening of a MACV vial meant health workers needed to ensure the number of children for a vial to avoid vaccine wastage or shortage. This led to strengthening the capacity of the health workforce, clear communication strategies in opening of vials, raising awareness of caregivers and engaging caregivers regarding the administration and delivery of the vaccine (Nkwenkeu *et al.*, 2020). Similarly, the RTS,S/AS01E vaccine demands adequate infrastructure to deliver its four doses.

The pre-introduction phase of the rotavirus and pneumococcal vaccines in Ghana is also a relevant example for the roll-out of the malaria vaccine because the introductory phase required resolving challenges, including limited cold-chain set-up, the logistics of moving the vaccine from one region to the other and community mobilization. According to the EPI, the rotavirus and pneumococcal vaccines were successfully introduced due to support received from GAVI, evidence-based data, expanding its cold-chain set-up, strengthening its health workforce and community mobilization and clear communication with caregivers on the health benefits (Donkor *et al.*, 2013; Enweronu-Laryea *et al.*, 2014b).

For the scale-up of the rotavirus and pneumococcal vaccines, policymakers went through the process of factoring both vaccines’ effectiveness, funding and cold-chain set-up and the cost-effectiveness of introducing the two vaccines. A similar process is likely to occur in Ghana with the scale-up of the RTS,S/AS01E malaria vaccine. However, there is an important difference between the two vaccines introduced simultaneously and this malaria vaccine: the previous vaccines were first introduced in other countries before the Ghana national immunization programme introduced them (PATH, 2013). The rotavirus vaccine and pneumococcal vaccine went through a lengthy pilot phase in other countries and for 1–2 months in Ghana to resolve unanswered safety questions (Donkor *et al.*, 2013; Enweronu-Laryea *et al.*, 2014b). The RTS,S/AS01E malaria vaccine, on the other hand, was introduced in Malawi, Ghana and Kenya as the first of its kind (Hogan *et al.*, 2020). Thus, it will need to be implemented on a longer time frame in Ghana before stakeholders can consider including it in the national immunization programme.

Moreover, results from Phase 3 trials on the feasibility, impact and safety of the malaria vaccine, especially its effectiveness, have revealed some concerns that need resolving (WHO, 2018b). Importantly, the efficacy of the RTS,S/AS01E malaria vaccine is not as high as the rotavirus vaccine and pneumococcal vaccine (Wilder-Smith *et al.*, 2017). However, it is still of public health relevance because a malarial infection can lead to other severe forms of diseases and deaths. All of the factors identified in this study in the context of the malaria vaccine were experienced previously by the EPI when introducing the rotavirus and pneumococcal vaccines (Donkor *et al.*, 2013; PATH, 2013; Enweronu-Laryea *et al.*, 2014b). Similar to the COVID-19 vaccines that require expansion of existing cold-chain system, the malaria vaccine will require the same (Tagoe *et al.*, 2021). Although this study provides a basis for assessing the facilitators and barriers, additional information gathering on a larger scale involving a more diverse group of stakeholders and vaccine experts to determine if the results can be replicated seems appropriate. Second, this could confirm if thematic saturation was achieved or if this represents an important starting point for additional exploration.

## Strengths and limitations of the study

This study is built on IDI analysis of stakeholders who have been involved in combatting the burden of malaria in Ghana for over three decades. Study participants were identified based on their extensive experience and their key national roles in combatting the impact of malaria. Furthermore, the inclusion of these study participants was an asset to the study because of their success in the implementation of past malaria interventions. Interviewees also drew on knowledge of factors that have been pivotal in the nationwide implementation of other previous vaccines in Ghana. The results from this qualitative study are not generalizable to other stakeholders or populations, but the results are similar to those from other studies conducted in Kenya, South Africa and Gambia on the introduction of the rotavirus and pneumococcal vaccines, and provide important considerations for planning the scale up of a vaccine (Roca *et al.*, 2011; Muendo *et al.*, 2018; Kleynhans *et al.*, 2021). In addition, the small sample size may not represent all views on the topic. This may have narrowed the scope of the data and information gathered. We did not interview other vaccine experts who may have also provided useful information. This study is not the concluding point but provides the basis for additional exploration regarding the barriers and facilitators on a larger scale study.

## Conclusion

This study identified key factors that may promote and inhibit the nationwide implementation of the RTS,S/AS01E malaria vaccine implementation and scale-up in Ghana. Ghana’s disease burden and current lack of vaccine availability are effective drivers for adding new vaccines to the immunization schedule, which currently hosts 13 antigens (WHO/UNICEF, 2017). The evidence-based data generated and gathered during the MVIP phase will be used to guide decisions, justifications and the cost-effective analysis of the policy. If the EPI, FDA, PATH, WHO and other institutions linked to the GHS can ensure feasibility, confer the ‘expected’ efficacy level, and no serious adverse events following immunizations, then policymakers may be more comfortable recommending the vaccine. Despite the partial efficacy of the RTS,S/AS01E malaria vaccine, the benefits of this vaccine are likely to be important in reducing the malaria cases and deaths among children up to aged 5 years in Ghana and other malaria-endemic countries. To increase the support of rolling out the malaria vaccine, the GHS must leverage the identified driving factors and strengthen the health system at both the district and regional levels to accommodate vaccines such as the malaria vaccine. The GHS should strengthen and provide a cold-chain set-up across the various levels of the peripheral health facilities. The GHS through the health promotion division should intensify public education about the efficacy of the vaccine throughout the country. Social mobilization is also needed to educate mothers or caregivers on the importance and acceptance of having children take all four doses, including promoting follow-up on taking the four doses.

The context of scaling up the vaccine should encompass communication and understanding of the comprehensive intervention approach to reduce vaccine hesitancy. Therefore, it is important to employ social and behaviour change communication (SBCC) (MOHFW, 2013). The SBCC should be targeted, participatory and designed with available evidence-based data from the MVIP to strengthen community engagement and improve vaccine acceptance. The nature of community engagement is likely to have an impact on the populace perception of the vaccine. Conversations on the feasibility, impact and safety of the vaccine could also be held to address the concerns of caregivers. This participatory action could potentially increase caregivers’ adherence to coming to health facilities with their children for the vaccine administration (WHO, 2018a). With the COVID-19 pandemic, it will be important to explore how the COVID-19 vaccine hesitancy is impacting the uptake of the malaria vaccine and how the pandemic may have disrupted the delivery of the malaria vaccine on the immunization schedule.

## Data Availability

Data used in this paper will be made available when a reasonable request is made to the corresponding author.

## Reflexivity statement

The authors include two females and three males representing different streams in public health with research experience in Africa, Asia, Europe and North America. Four of the authors have expertise in maternal and child health, air pollution studies and health impact analysis in Africa, Asia and Europe. One of the authors has expertise in vaccine implementation programmes in Africa, and another has extensive experience in sociology. Three authors have experience conducting qualitative and interventional studies in Ghana.
